# Detecting proliferation of adult hemocytes in *Drosophila* by BrdU incorporation and PH3 expression in response to bacterial infection

**DOI:** 10.12688/wellcomeopenres.14560.2

**Published:** 2018-08-15

**Authors:** Saikat Ghosh, Sudip Mandal, Lolitika Mandal

**Affiliations:** 1 Developmental Genetics Laboratory, Department of Biological Sciences, Indian Institute of Science Education and Research-Mohali, Manauli, Punjab, 140306, India; 2 Molecular Cell and Developmental Biology Laboratory, Department of Biological Sciences, Indian Institute of Science Education and Research-Mohali, Manauli, Punjab, 140306, India

**Keywords:** Adult Drosophila, Hematopoiesis, Proliferation, Macrophage, Infection

## Abstract

*Drosophila* and mammalian hematopoiesis share several similarities that range from primitive and definitive phases of hematopoiesis  to the battery of transcription factors and signaling molecules that execute this process. The similarities in blood cell development across these divergent taxa along with the rich genetic tools available in fruitfly makes it a popular invertebrate model to study blood cell development both during normal and aberrant scenarios.

The larval system is the most extensively studied till date. Several studies have shown that these hemocytes just like mammalian counterpart proliferate and get routinely regenerated upon infection. However, employing the same protocol it was concluded that blood cell proliferation although abundant in larval stages is absent in adult fruitfly.

The current protocol describes the strategies that can be employed to document the hemocyte proliferation in adulthood. The fact that a subset of blood cells tucked away in the hematopoietic hub are not locked in senescence, rather they still harbour the proliferative capacity to tide over challenges was successfully demonstrated by this protocol.  Although we have adopted bacterial infection as a bait to evoke this proliferative capacity of the hemocytes, we envision that it can also efficiently characterize the proliferative responses of hemocytes in cancerous conditions like leukemia and solid tumors as well as scenarios of environmental and metabolic stresses during adulthood.

## Introduction

*Drosophila* hematopoiesis generates three kinds of mature differentiated blood cell types: plasmatocytes, crystal cells, and lamellocytes which are responsible for phagocytosis, melanization and encapsulation respectively ( Crozatier & Meister, 2007; Evans *et al*., 2003; Lebestky *et al*., 2000; Meister, 2004; Parsons & Foley, 2013). Amongst the three, plasmatocytes are the major cell type which acts as “professional macrophages” and consists of ~95% of total hemocyte population. Detail investigations on the behaviour of *Drosophila* macrophages revealed that they are multitaskers ( Brückner *et al*., 2004; Bunt *et al*., 2010; Dimarcq *et al*., 1997; Evans *et al*., 2010; Franc *et al*., 1996; Tucker *et al*., 2011; Wood *et al*., 2006). In all developmental stages they are actively involved in tissue morphogenesis (by secreting ECM proteins), engulfment of apoptotic cells, incessant surveillance against foreign infection and wound healing ( Wood & Jacinto, 2007). During immune surveillance, along with phagocytosis of pathogen, the hemocytes also secrete antimicrobial peptide (AMP) to combat infection ( Dimarcq *et al*., 1997). Hemocytes play a crucial role in inter-organ communication by secreting cytokine-like Unpaired (Upd) to activate JAK/STAT signalling as well as Spatzle (Spz), a pro-inflammatory cytokine to activate Toll signalling in fat body for AMP production ( Agaisse *et al*., 2003; Kounatidis & Ligoxygakis, 2012; Shia *et al*., 2009). The embryonic hemocytes after egg hatching divide and populate the larval hemolymph. These macrophages switch to a proliferative state in the larval circulation and increase their number ( Holz *et al*., 2003; Leitão & Sucena, 2015; Makhijani *et al*., 2011). In addition to the above population, larval hemocytes migrate into specific immunological sites present in the dorsal and lateral side of larval cuticle ( Makhijani *et al*., 2011; Makhijani *et al*., 2017; Márkus *et al*., 2009). The circulating hemocytes are guided by peripheral nervous system to home into the hematopoietic pockets where activin-β signaling regulates their proliferation ( Makhijani *et al*., 2011; Makhijani *et al*., 2017). This proliferative property of plasmatocytes, apart from helping them to increase their number, facilitates their transdifferentiation to lamellocytes during wasp infection ( Anderl *et al*., 2016).

Interestingly, a separate source of larval hemocyte arises from the *Drosophila* blood-forming organ: lymph gland. Within this organ, the hemocytes proliferate and increase the organ size during larval development ( Jung *et al*., 2005). At the time of pupation, the mature lymph gland ruptures and releases the hemocytes into circulation ( Grigorian *et al*., 2011; Jung *et al*., 2005; Lebestky *et al*., 2000). Although proliferation in hemocytes is in abundance in immature stages, it is not detectable in the adult fruit fly. This observation led to the proposition that adult hemocytes are in a state of senescence ( Honti *et al*., 2014).

The identification and characterization of Hematopoietic Hub in *Drosophila* ( Ghosh *et al*., 2015; Ramond *et al*., 2015) clearly revealed that the process of new blood cell formation continues even in the adult stage and it seems to be relevant for combating microbial infection. The adult hematopoietic hub, positioned at the dorsal side of the heart tube houses hemocytes nested in an intricate network of extracellular matrix proteins like Pericardin and Laminin A. Hemocytes from both embryonic and larval lineages home into this dorsal abdominal site. In addition to differentiated cells homing in, several hematopoietic progenitor cells arrive in the hub, which subsequently employs Notch signaling to differentiate into mature plasmatocytes and crystal cells. Moreover, the study also demonstrated that the adult macrophages which were thought to be in senescence can still proliferate in response to a high dose of infection ( Ghosh *et al*., 2015). Here, we describe a detailed step-by-step protocol to assay the proliferative capacity of adult hemocytes by BrdU incorporation and PH3 expression in response to high dose of bacterial infection.

## Methods

### Reagents

- 1X Phosphate buffered saline (1X PBS)
- 5-bromo-2'-deoxyuridine (BrdU) (Sigma Cat. No. B5002)
- Paraformaldehyde (Sigma Cat. No. P6148)
- Sodium Deoxycholate (Sigma Cat. No. D6750)
- TritonX 100 (Merck Cat. No. MB031)
- Bovine serum albumin (BSA) (Sigma Cat. No. A7906)
- Sodium azide (Sigma Cat. No. S2002)
- Hydrochloric acid (HCL) (Merck Cat. No 101834)
- Colchicine (Sigma Cat. No. C9754)
- Phospho Histone H3 (Cell Signalling, Cat. No. 9713, RRID: AB_823532)
- Monoclonal Anti-Green Fluorescent Protein (GFP) antibody produced in mouse clone GFP-20, ascites fluid (Sigma Cat. No. G6539, RRID: AB_259941)
- Rat monoclonal anti-BrdU antibody [BU1/75 (ICR1)], (Abcam Cat. No ab6326, RRID: AB_305426)
- FITC conjugated Goat anti-mouse antibody (Jackson Immuno Research Cat. No. 115-095-166, RRID AB_2338601)
- Cy3 conjugated Donkey anti-rat antibody (Jackson Immuno Research Cat. No. 712-165-153, RRID: AB_2340667)
- Cy3 conjugated Donkey anti-rabbit antibody (Jackson Immuno Research Cat. No.711-165-152)
- Vectashield (Vector Laboratories, Cat. No. H-100)
- *HmlGal4.UAS GFP* flies (BDSC Cat# 30140, RRID:BDSC_30140)
- *Escherichia coli* (DH5α) expressing RFP (pFPV25.1 RFP plasmid)

### Equipment

- Fly food vials
- Dissecting microscope (Carl Zeiss Stemi 2000)
- Orbital shaker (New Brunswick Scientific Excella E5 Platform Shaker)
- Glass slide (Blue Star Micro Slides PIC-1, size: 75X25mm, thickness: 1.35mm)
- Cover glass (Blue Star Microscopic Cover Glass, Size: 24X24mm)
- Glass capillaries (Sutter Instruments)
- Fine surgical Forceps (Local surgical store)
- Fine surgical Scissors (Local surgical store)
- Dissecting needles
- Pipetman
- Pipette tips
- Moist chamber
- Nail polish
- 4°C Refrigerator
- Confocal microscope (Carl Zeiss LSM 780 & Leica SP8)
- Imaging software ( Image J, RRID:SCR003070, Photoshop CS3. RRID: SCR002078 and Bitplane Imaris 64X, RRID: SCR007370)

***BrdU feeding of adult fly.*** To assay the cell proliferation in adult hemocytes, a synchronized collection of freshly eclosed adult flies is collected. The feeding protocol is adapted and further modified from the study ( Micchelli & Perrimon, 2006). ( *A minimum number of 40-adult flies per set of experiment is used*).

1. 3 ^rd^-day old adult flies ( *+;hmlGal4-UASGFP;+*) are transferred from normal food to cornmeal yeast fly food (2ml volume) which is supplemented with 200μl of 6mg/ml BrdU. *(BrdU solution freshly prepared in PBS)*.
2. The flies are then reared for two days in the BrdU containing food. [Incorporation of Bromodeoxyuridine (BrdU), a thymidine analog is an established assay for determining cell proliferation in different organisms. Replicating cells during their S phase of the cell cycle readily incorporates BrdU instead of thymidine. ( *Rearing flies in BrdU food generates a pool of BrdU in the system that increases the chance of incorporation in cells especially the ones that undergo rare cell division).* The BrdU incorporation can be detected by commercially available anti-BrdU antibody].
3. Post two days of rearing in the BrdU supplemented food, flies are infected with *E.coli* by following the procedure as mentioned below.

***Bacterial infection of the adult fly.***

**Figure 1.**
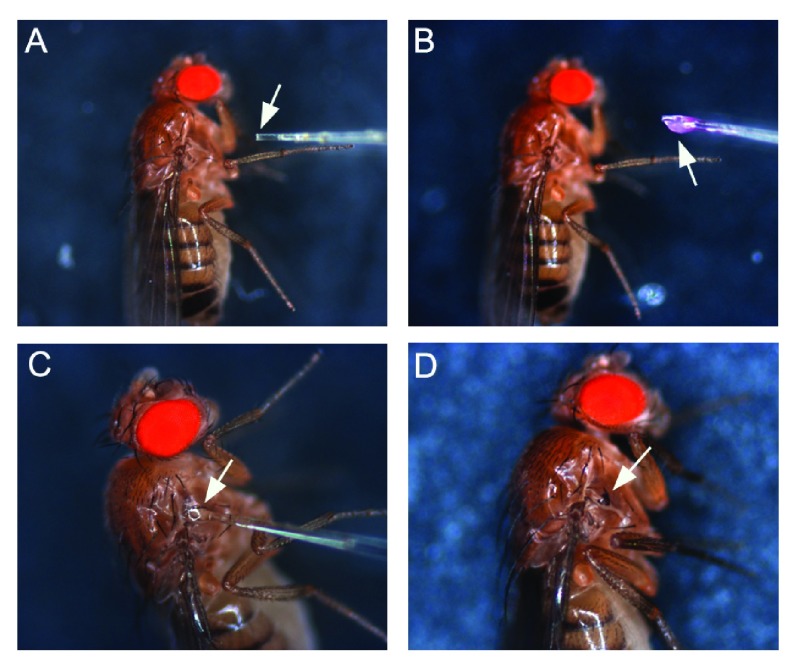
Infection procedure in adult *Drosophila*. ( **A**) A fine glass capillary with a pointed tip (arrow) is used to infect an anesthetized adult fly. ( **B**) The image shows a colony of RFP expressing *E.coli* picked up at the tip of the capillary (arrow). ( **C**) The lateral side of the thoracic region is pierced with the fine capillary. Arrow shows the position at which the capillary is inserted in the thorax. ( **D**) 24 hour post infection; the site of insertion in the thorax can be detected by the presence of a black melanised spot.

1. The sharp end of the capillary is dipped in a colony of *Escherichia coli* expressing RFP (pFPV25.1 RFP plasmid) ( Figure 1B).
2. Using this fine glass capillary, lateral side of the thorax of a pre-anesthetized adult fly is pierced ( Figure 1A–D).
3. Infected flies are re-transferred in BrdU supplemented food and reared for five days before dissection. In between two intermittent flips are required in fresh BrdU food. *[Post infection BrdU feeding is important to maintain the level of BrdU, which increases the chance of incorporation in the slowly dividing hemocytes in response to bacterial infection. Two consecutive flips in fresh BrdU food ensures a constant source of BrdU and maintains healthy fly culture*]
4. The flies are dissected and immuno-stained using anti-GFP (to mark the hemocytes: *hml-GFP*), and anti-BrdU antibody to visualize the replicating cells. Co-localization of GFP and anti-BrdU antibody expression will ensure that the replicating cell is indeed a hemocyte.

As a control experiment, a mock infection was performed. The thorax of adult flies are pierced with fine glass capillary dipped in sterile 1X PBS (similar to point 2) and the flies are then subjected to all the steps similar to the experimental flies.

***Adult fly dissection for Hematopoietic Hub isolation.*** BrdU fed adult flies are anesthetized and dissected carefully using fine scissors and forceps in ice-cold 1X PBS (pH 7.2).

**Figure 2.**
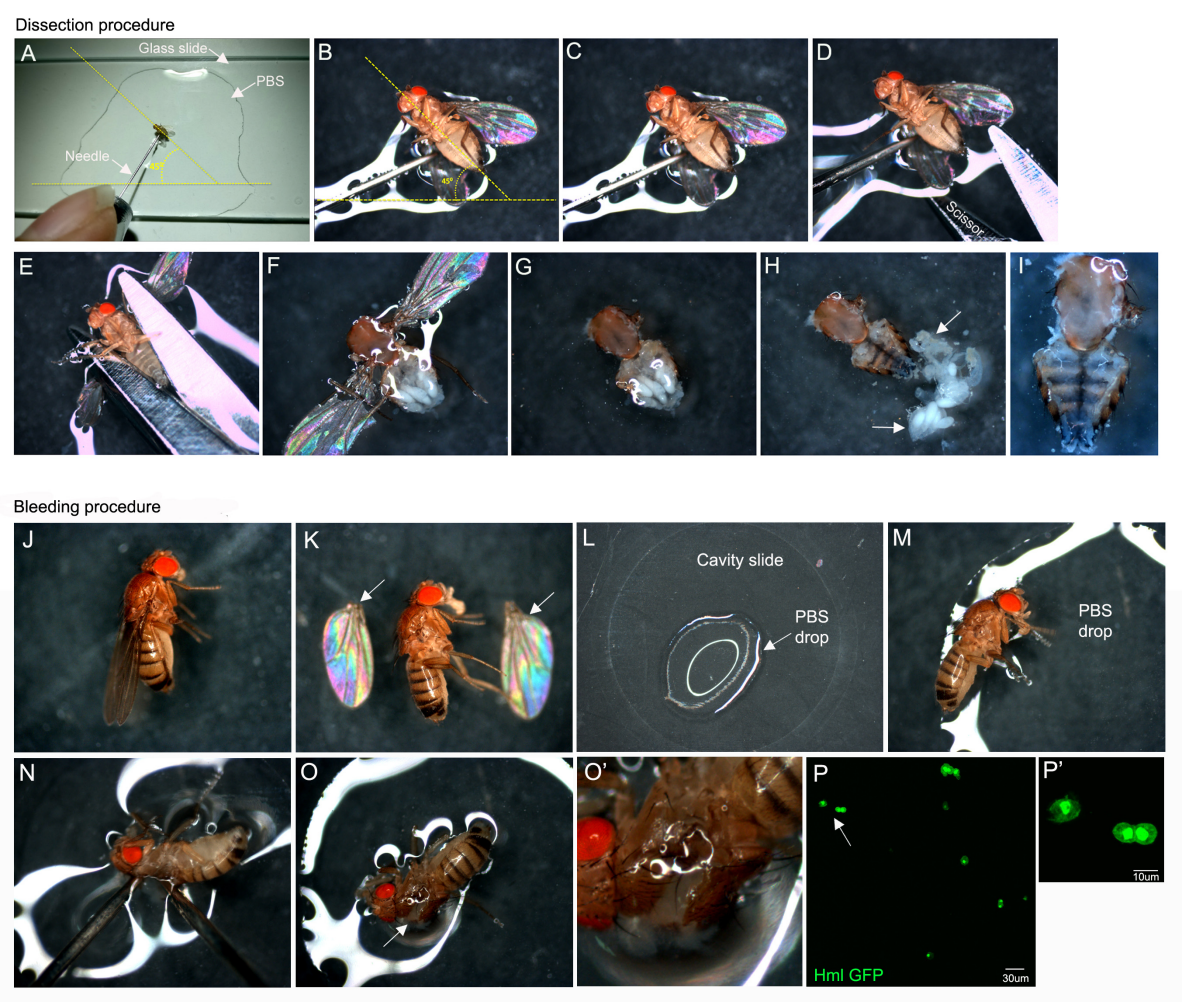
Adult fly dissection procedure to isolate the hematopoietic hub and adult fly bleeding procedure. ( **A**) The anesthetized adult fly is placed on a clean glass slide at an angle of 45° (held by a dissecting needle). ( **B**– **C**) The higher magnification shows the exact position of the adult fly. The fly wings are stretched apart so that the dorsal surface of the abdomen is submerged in the PBS solution. ( **D**) A fine surgical scissor is placed parallel to the fly and ( **E**) An incision is made with the scissor from the posterior tip of the anus and vaginal plate running ventrally to the anterior end of the fly. ( **F**) The ventral side of abdomen, legs and the head are removed carefully. The dissected sample consists of only the dorsal side of the thorax and the abdomen ( **G**) The wings are removed with the help of needles. ( **H**) The tissues (gut, ovaries, malpighian tubules) present inside the abdomen are gently removed (arrow) without disturbing the dorsal diaphragm. ( **I**) Once the dissection is complete, the sample containing the dorsal side of the thorax and exposed abdomen is processed for immunostaining. ( **J**) Anesthetised adult fly is placed on a clean glass slide. ( **K**) The wings (arrow) are removed with the help of scissor. ( **L**) A drop of 20μl PBS is placed inside a cavity slide. ( **M**) The fly from **K** is now placed in the drop of PBS. ( **N**) The thorax is opened with the two fine needles. ( **O**) Hemolymph oozes out from the exposed region of the thorax and gets collected in the drop of PBS. ( **O’**) Higher magnification of ( **O**) shows the exposed thoracic region. ( **P**– **P’**) A snapshot of the bleed containing Hml GFP expressing hemocytes.

1. The flies are positioned dorsal side down in a drop of PBS placed on a clean glass slide ( Figure 2A).
2. Using two needles, the wings are stretched apart so that the dorsal abdomen comes in contact with the PBS surface ( Figure 2B–C). It is very important to ensure that dorsal half of the abdomen is submerged in PBS. [ *This step is crucial as the wings are hydrophobic in nature, therefore, they obstruct the dorsal abdomen of the fly from dipping into the PBS*].
3. A clean scissor, rinsed in alcohol, is used for dissecting the fly from ventral side ( Figure 2D–E) ( *Dissection is done from the ventral side as the hematopoietic hub is positioned on the dorsal side of the abdomen*).
4. The fly is placed at an angle of 45°. In order to hold the fly in that position, a dissecting needle from one side is placed on the stretched wing ( Figure 2A–C) and a scissor is aligned parallel to the fly (as shown in Figure 2D). A fine incision is then made from the posterior tip of the fly abdomen ventrally *(i.e. anus and vaginal plate)* and continued up to the head ( Figure 2E). While doing so, care should be taken to ensure that the pale non-pigmented ventral part of the abdomen is detached from rest of the fly.
5. During this procedure, the head automatically gets disengaged from the rest of the body. The dissected fly body part consists of the dorsal side of the thorax and abdomen ( Figure 2F–G).
6. The wings are then removed with help of a sharp scissor. While the thorax is retained for holding the tissue for the entire duration of the staining procedure. *[This ensures that the hub hemocytes in the abdomen remain undisturbed]*.
7. The tissues present inside the abdominal cavity (like gut, ovaries, malpighian tubules) are gently removed while the dorsal abdominal diaphragm is kept untouched ( Figure 2H). This dorsal abdominal diaphragm composed of fat body layers, which are tightly connected with alary muscles, heart muscle, and pericardial cells. All of these in some way contribute to maintain the structural integrity of the Hub. *[Therefore, in this entire process of dissection, it has to be ensured that the sharp ends of needles and scissors should not poke the dorsal side of the abdomen. Otherwise, this might dismantle the Hematopoietic Hubs, thereby interfering with the analyses]*.
8. The samples are now ready for immunostaining ( Figure 2I).

***Immunostaining of adult hematopoietic hub***. *Please note all incubation and washings are done at room temperature unless otherwise mentioned.*

1. The dissected samples are fixed in freshly prepared 4% para-formaldehyde for 45 min in 1X PBS on a shaker.
2. Post fixation the samples are washed thrice in 1X PBS for 10 min each followed by 30 min incubation in 0.3%PBT + 0.3% Sodium Deoxycholate on a shaker (0.3%PBT = 0.3% TritonX 100 in 1X PBS.) *[Both Triton X100 and Sodium Deoxycholate are used for permeabilization of the membrane*].
3. The permeabilized tissues are incubated in freshly prepared blocking solution (10% bovine serum albumin [BSA] in 1X PBS) overnight at 4°C.
4. Post blocking, samples are incubated in primary antibody (mouse anti-GFP, 1:50) diluted in 1X PBS for 45hr at 4°C (primary antibody is supplemented with 1µl of 0.02% Sodium azide). [Addition of *sodium azide reduces the chance of infection during this long duration of antibody incubation*].
5. Samples are then washed thrice in 1X PBS for 10 min each and subsequently incubated in blocking solution on a shaker.
6. The blocking solution is replaced by Secondary antibody (anti-mouse FITC, 1:400, diluted in 1XPBS) and the tissues are incubated for 45hr at 4°C. (Henceforth, all incubations are carried out in a dark chamber).
7. Three washes in 1X PBS of 10 min each are done post secondary antibody incubation.
8. 1X PBS is aspirated out and the tissues are briefly treated with 0.3%PBT + 0.3% Sodium deoxycholate for 15 min [ *The brief wash ensures efficient permeabilization*]. This step is followed by a wash in 1X PBS for 10 min to remove the detergents.
9. The tissues are then re-fixed with 4% para-formaldehyde for 20 min on a shaker.
10. Post-fixation samples are washed thrice with PBS for 10 min each.
11. For depurination step, PBS is replaced by 2N HCl (freshly prepared in PBS) and the sample is first incubated for 10 min on a shaker followed by 30min incubation without shaking. [ *HCl treatment denatures the DNA and thus allows the anti-BrdU antibody access to the BrdU within the DNA. As HCl treatment is harsh for the cells, therefore, they were subjected to a minimum shaking time.*].
12. Traces of HCl is removed by one quick and two 10min washes in 1X PBS.
13. Samples are then blocked in 10% BSA for an hour followed by incubation in rat anti-BrdU primary antibody (1:100, prepared in 1X PBS) for 45hr at 4°C.
14. Three washes in PBS are done before adding the secondary antibody (anti-rat Cy3, 1:400) for 45hr at 4°C.
15. Post incubation two gentle PBS washes of 10 min each are done.
16. To visualize the nucleus of cells, the tissues are incubated in DAPI solution (prepared in 1X PBS) at 4°C for overnight followed by washing twice in PBS (10 min each).
17. Finally, samples were mounted in mounting media (Vectashield).

***Colchicine feeding of adult fly and PH3 staining***. Colchicine is known to interfere with cell division by inhibiting microtubule polymerization and therefore an exposure to it is sufficient enough to arrest cells in mitosis ( Taylor, 1965; Viktorinová & Dahmann, 2013).

*To assay the rare event of adult hemocyte proliferation by Phospho-histone 3 expression, colchicine is administered through food. A suitable exposure to colchicine induces accumulation of metaphase-blocked mitoses. Phospho-histone H3 (PH3), an immunomarker specific for cells undergoing mitoses was employed to track the proliferative hemocyte.*

*Colchicine administration results mitotic arrest that facilitated the chances of marking the proliferative hemocytes with PH3, post bacterial infection.*

*A synchronised collection of freshly eclosed flies are collected.*

1. 5 ^th^ day old adult flies (+ *;hmlGal4-UASGFP;*+) are transferred from normal food to cornmeal yeast fly food (2ml volume) which is supplemented with freshly prepared Colchicine (in water, final concentration 1mg/ml in food).
2. The flies are reared for 6hrs in this Colchicine containing food. [ *Colchicine is an alkaloid which blocks the microtubule polymerisation in cells and thus arrests the dividing cells in different stages of mitosis]*
3. Flies are then infected with *E.coli* by following the procedure as mentioned above section ‘ *Bacterial infection of adult fly’*.
4. Infected flies are re-transferred in Colchicine supplemented food and reared for 18hrs before dissection. *[Post infection Colchicine feeding is important to maintain the level of Colchicine which increases the chance of mitotic arrest of these rarely dividing hemocytes in response to bacterial infection.*]
5. The flies are dissected and immuno-stained by following the above-mentioned section *‘Immunostaining of adult hematopoietic hub’* (follow point number 1-7) where primary and secondary antibody used anti-PH3 antibody, 1:100 and anti-rabbit Cy3, 1:400 respectively (to mark *phospho-histone 3 expression*). Co-localization of *hml GFP* and anti-PH3 antibody expression ensured that the cell is certainly a proliferating adult hemocyte.

***Mounting of adult fly samples.*** The mounting steps of the adult abdominal samples are critical for successful observation of adult Hematopoietic Hub. Before mounting, trimming of the cuticle on either side of the abdomen is required. This cuticle along the edges otherwise attributes unwanted thickness to the tissue.

1. A drop of 1X PBS is taken on a clean glass slide. As described previously in the “dissection” section, the sample is placed at an angle of 45° and thorax and the curved cuticle is removed by applying a very sharp cut along the extreme edge of the abdomen where the curvature initiates.
2. After trimming both sides of the abdomen, the thorax is removed very carefully. [ *The 1 ^st^ hematopoietic hub along with the conical chamber of the heart is tightly associated with a thoracic-abdominal junction, therefore this step is extremely crucial*].
3. Samples are next transferred on a clean slide containing a drop of Vectashield and incubated for 15–20 min. [ *Incubation of samples inside dense Vectashield is an essential step to reduce the opacity observed due to aqueous layer associated with a thick layer of the fat body which makes deep tissue imaging under the microscope a bit challenging*].
4. Finally, the tissues are arranged in a row and a cover-slip is gently placed on them. In order to prevent drying, edges of the cover slip is sealed with a transparent nail-polish. The mounted samples are ready for immediate imaging or can be stored in 4°C.

***Fly Bleeding.***

1. To assay the cell proliferation in adult circulatory hemocytes, a batch of synchronously eclosed adult flies are reared in BrdU containing food before and after the bacterial infection, following the above-mentioned procedure (Section: ‘ *BrdU feeding of adult fly*’).
2. The synchronized culture of ( *+;hml Gal4- UAS GFP;+*) female flies are anesthetized and the wings are removed using fine scissors ( Figure 2J–K).
3. The fly is then placed in a drop of 1X PBS (20μl) on a clean, pre chilled glass cavity slide ( Figure 2L–M). [ *Chilled glass slide minimizes the evaporation of the small amount of PBS*]
4. With the aid of two needles, a fine incision is made on the lateral side of the thorax without disturbing the adult abdomen proper. The exposed region of the thorax is carefully positioned in the drop of PBS for 20 seconds in order to collect the hemolymph along with blood cells ( Figure 2N–P’). ( *A total of 8 flies are taken to bleed in a drop of 20μl PBS).*
5. The hemocytes are allowed to adhere to the glass surface for 20 min inside a moist chamber.
6. Fixation of the hemocytes is done by adding freshly prepared 4% para-formaldehyde for 30min inside a moist chamber.
7. After three washes in 1X PBS for 5min each, 0.3%PBT + 0.3% Sodium Deoxycholate is added on the hemocytes for 5 min. [ *For permeabilization of the membranes*]
8. Sodium Deoxycholate is removed by two washes in 1X PBS for 10min each.
9. The cells are then incubated in the blocking solution (10% bovine serum albumin (BSA) prepared in 1X PBS) for 30min.
10. The blocking solution is replaced by the primary antibody (mouse anti-GFP, 1:50, diluted in 1X PBS). Incubation is done for 18hr at 4°C. Cells are then washed thrice in 1X PBS for 5min each.
11. Secondary antibody (anti-mouse FITC, 1:400) incubation is carried out in dark chamber, for 4hr at RT followed by three 10 min washes in 1X PBS.
12. For depurination step, 2N HCl (freshly prepared in 1XPBS) is added for 20 min without shaking.
13. Two washes in 1XPBS for 10min each is next done to remove traces of HCl.
14. The samples are next incubated in rat anti-BrdU primary antibody (1:100), prepared in 1X PBS for 4hr.
15. Post incubation, two washes in 1XPBS for 10min is carried out before blocking it with 10% BSA block for 30min.
16. Secondary antibody anti-rat Cy3 (1:400) is added and incubated for 4hr. This was followed by two-three washes in PBS for 10min each.
17. The nucleus of cells is next labeled with DAPI. (A incubation in DAPI solution for 10min followed by two washes of 10 min each in 1X PBS was done).
18. Samples are mounted in mounting media Vectashield for immediate imaging.

***Imaging.***

1. The circulating hemocytes or hematopoietic hub are imaged using a combination of laser lines 405nm, 488nm, and 561nm in a confocal microscope.
2. Images are further processed in ImageJ and Photoshop software. In ImageJ, the raw confocal data file (.lsm/.LIF) are opened and visualized the combined image (Image>Color>Make composite). Then each channel are analysed by following Image>Color>Channel tool option. Brightness/contrast are adjusted in equal level both in control and experiment following Image>Adjust>Brightness/Contrast. Finally, the images are converted to RGB (Image>Color>Stake to RGB) and saved in TIFF format (File>Save as>Tiff). Photoshop software is used to arrange and prepare the high-resolution figure panels.
3. 3D surface rendered models are generated using Imaris 64X software (7.6 version).

The steps below were followed:

a. In Imaris, the raw confocal data of multiple Z stacks are opened and visualised in ‘Surpass’ mode.
b. Open ‘Display Adjustment’ window to analyse each color channel (GFP, Cy3, DAPI).
c. Next, click on to the ‘Surface’ option from the ‘Volume properties’ in the menu bar and follow the subsequent three steps (of the algorithm) to create the 3D surface model.
d. Next step ‘Source Channel’: select the Channel (GFP/Cy3/DAPI) and click ‘smooth’ and ‘absolute intensity’.
e. After that is ‘Threshold’: in this step effort has to be made to ensure that the selected object is perfectly covered by the newly created 3D surface. This depends on the volume observed from the respective fluorescence intensity of the object present in the image file.
f. Next is ‘Classify surface’: in this step click and finalise the 3D surface. After finalising the 3D surface selection click on to ‘Color’ panel and subsequent adjustment of the colour tone, transparency, intensity in the multicolour 3D model can be done according to the interest.
g. Finally, for saving the 3D model as single image select the ‘Snapshot’ and for 3D rotation select ‘Animation’ tool.

## Results

### Response against infected *E.coli* in adult hemocytes

**Figure 3.**
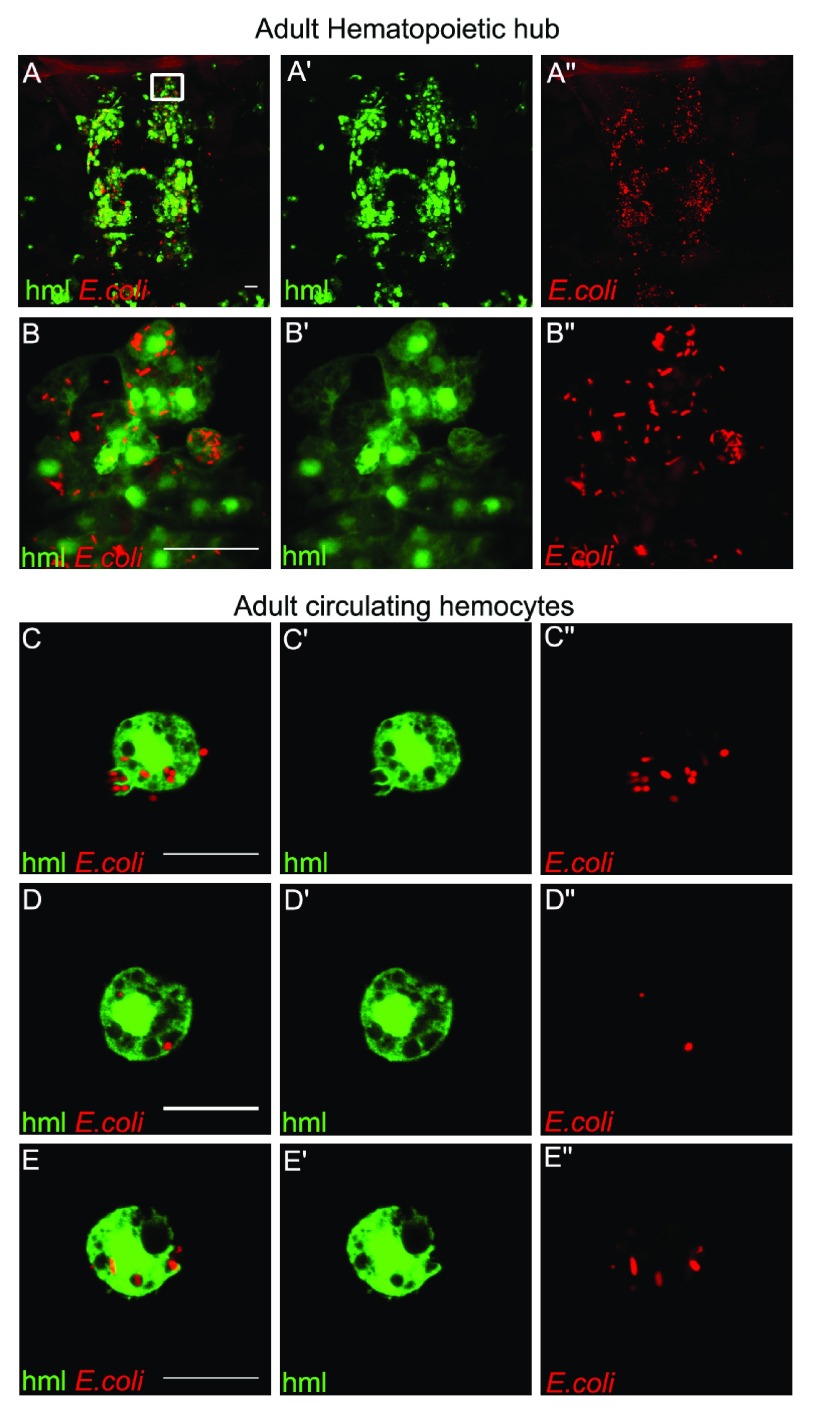
Phagocytic response against infected *E.coli* in hemocytes of hematopoietic hub and circulation. ( **A**– **B”**) 30 minutes post infection the hub hemocytes (green) shows a strong phagocytic response against the bacteria (red). ( **B**– **B”**) Higher magnification of **A** shows majority of the hemocytes has engulfed *E.coli.* Image shows multiple *E.coli* is engulfed by a single hemocyte present in the hematopoietic hub. ( **C**– **E”**) Multiple examples of circulating hemocytes engaged in phagocytosis of *E.coli* (red. ( **C**– **C”**) The hemocyte throws filopodial extensions to form phagocytic cup around the *E.coli* prior to phagycytosis. Scale bar: 20µm ( **A**– **B”**), 10µm ( **C**– **E”**).

***A. Phagocytic Response.*** Upon 30 minutes post infection with *E. coli,* the resident hemocytes of the hematopoietic hub are seen actively engaged in phagocytosis of the invading bacteria ( Figure 3A–B"). Instances can also be documented where multiple bacteria are phagocytosed by single hemocyte illustrating their rapid response to clear the huge infection load ( Figure 3B–B").

Similar behavior is seen from the circulating hemocytes ( Figure 3C–E"). Here also, single hemocytes can be seen throwing multiple filopodial extensions to engulf several *E. coli* cells from the infected hemolymph.

***B. Proliferative response.*** Previous attempts to evaluate the cell division potentiality of adult hemocytes, primarily involved infecting the flies and then subjected them to proliferation assay with BrdU. These assays followed normal protocol, which is generally employed to assay proliferation in eye disc or lymph glands ( Escudero & Freeman, 2007; Grigorian *et al*., 2011; Jung *et al*., 2005). In majority of these methods, dissected tissues are incubated in BrdU solution for 30–60 min so that any cell division happening at that time of incubation will incorporate the modified nucleotide source thereby getting labeled. Employing this strategy no division was detected in plasmatocytes, although, BrdU incorporation was evident in endo-replicating fat cells of both the control and infected adult flies.

**Figure 4.**
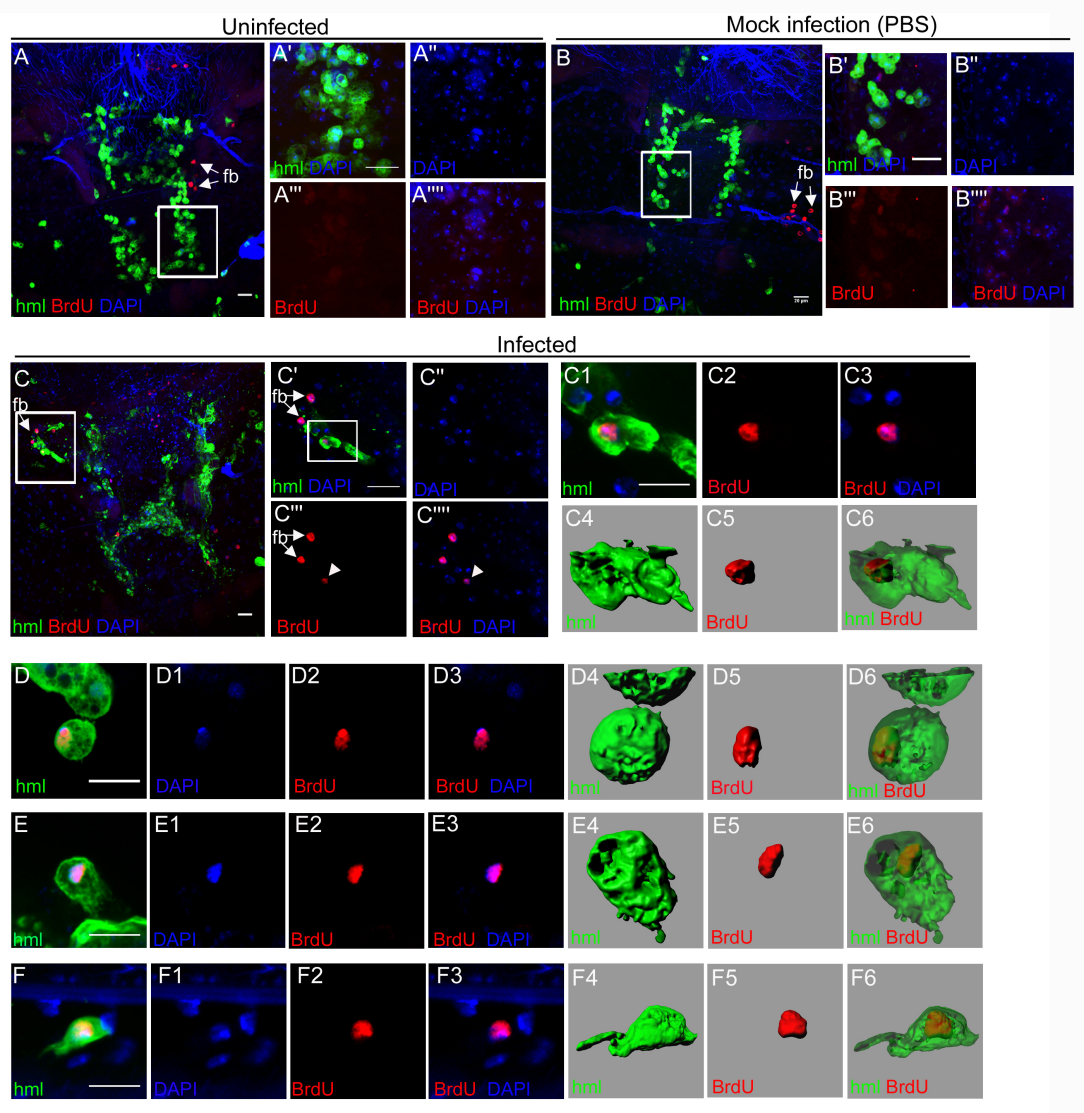
In response to bacterial infection hub hemocytes undergo proliferation. ( **A**– **A””**) In wild-type, adult fly hemocytes do not incorporate BrdU, while endo-replicating fat body (fb, arrow) cells incorporate BrdU and serves as an uninfected control. ( **B**– **B””**) Mock infection control set also does not show BrdU incorporation in the hemocytes, fat body cells showing BrdU signal serves as control tissue (arrow). ( **C**– **C””**) Infected individuals show BrdU incorporation in hub resident hemocytes indicating their proliferative state in response to infection. Higher magnification of C shows hml GFP ^+^ BrdU ^+^ hub hemocyte (arrowhead) juxtaposed to BrdU positive fat body cells ( **C’**– **C””**). ( **C1**– **C6**) BrdU incorporation in the hemocyte visualized in a single-cell resolution ( **C1**– **C3**) and 3D surface rendering (of **C1**) reveals the BrdU signal is present inside the hemocyte. ( **D**– **F6**) Transparent 3D surface model of individual hub hemocytes shows incorporation of BrdU in nucleus respectively. Scale bar: 20µm ( **A**– **C””**), 10µm ( **C1**– **F6**).

Employing the current method, no BrdU incorporation within the hub resident hemocytes is seen in an uninfected individual as well as in mock infection. Although the fat body cells (arrow in Figure 4A,B) which are endo-replicating in nature positively incorporated BrdU ( Figure 4A–B"").

In contrast, the infected flies showed several BrdU labeled plasmatocytes inside the hematopoietic hub along with nearby fat cells (arrowhead in Figure 4C–C""). The BrdU incorporation was specific to plasmatocyte nucleus was further confirmed by constructing 3D surface modeling at a single cell resolution ( Figure 4 C1–F6 and Supplemental Movie S1 and Supplemental Movie S2.

**Figure 5.**
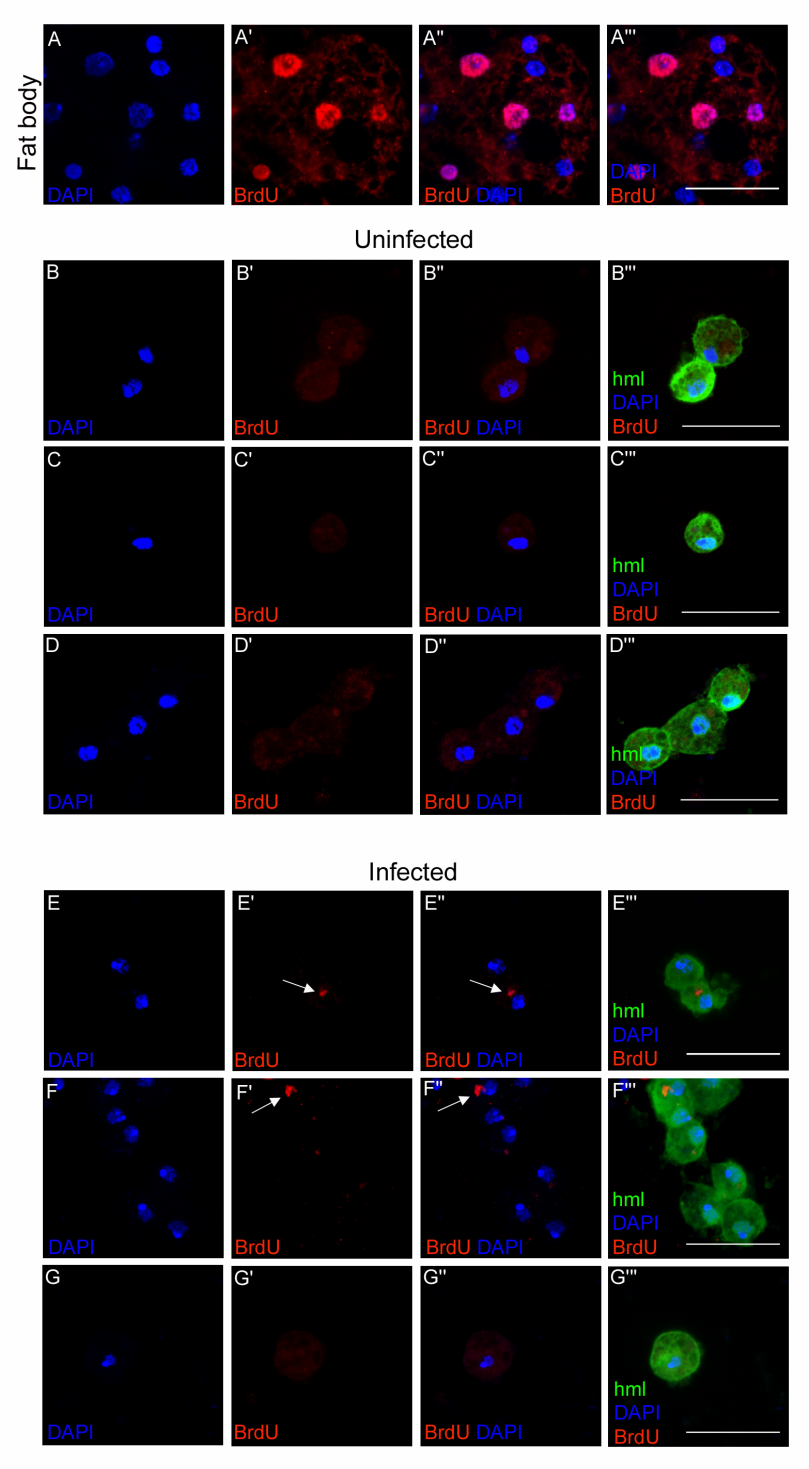
Circulating hemocytes do not show any proliferation. ( **A**– **A’”**) The adult endoreplicating fat body cells act as a positive control as they show BrdU (red) incorporation. ( **B**– **D’”**) The uninfected circulating hemocytes of adult flies are in their non-dividing state thus does not show any BrdU incorporation. ( **E**– **G’”**) Circulating hemocytes from *E. coli* infected Adult fly do not show any BrdU positive cells. Some hemocytes in this figure still retains some *E.coli* cells (tiny red dots present outside nucleus). Scale bar: 20µm.

However, employing the similar infection regime and BrdU assay the circulating hemocytes in adult fruit fly failed to incorporate BrdU ( Figure 5). While the fat body cells served as positive control for successful BrdU labeling ( Figure 5A–A"'), hemocytes from both uninfected ( Figure 5 B–D"') and infected ( Figure 5E–G"') individuals lacked any incorporation.

The hemocyte proliferation in the hematopoietic hub of the adult was further validated by another independent assay of cell proliferation, the Phospho-histone 3 (PH3) labeling which marks the mitotically active cells only ( Aune *et al*., 2011; Ribalta *et al*., 2004; Sawicka & Seiser, 2012). The BrdU incorporation assay revealed that the hemocytes undergoing replication in the adult are very less number. In addition to this, the event of replication seems to be random ( Ghosh *et al*., 2015). Therefore, to trace a mitotically active hemocyte in adult fly post-immune challenge is technically difficult. Considering the less duration of M phase compared to S phase in the cell cycle, a strategy was adopted to mark this rare mitotic phase of adult hemocytes. The adult flies were reared in food supplemented with colchicine (1mg/ml) to arrest the rarely dividing cells in mitosis thereby increases the probability of marking the mitotically active hemocytes upon bacterial infection (by PH3 labeling).

**Figure 6.**
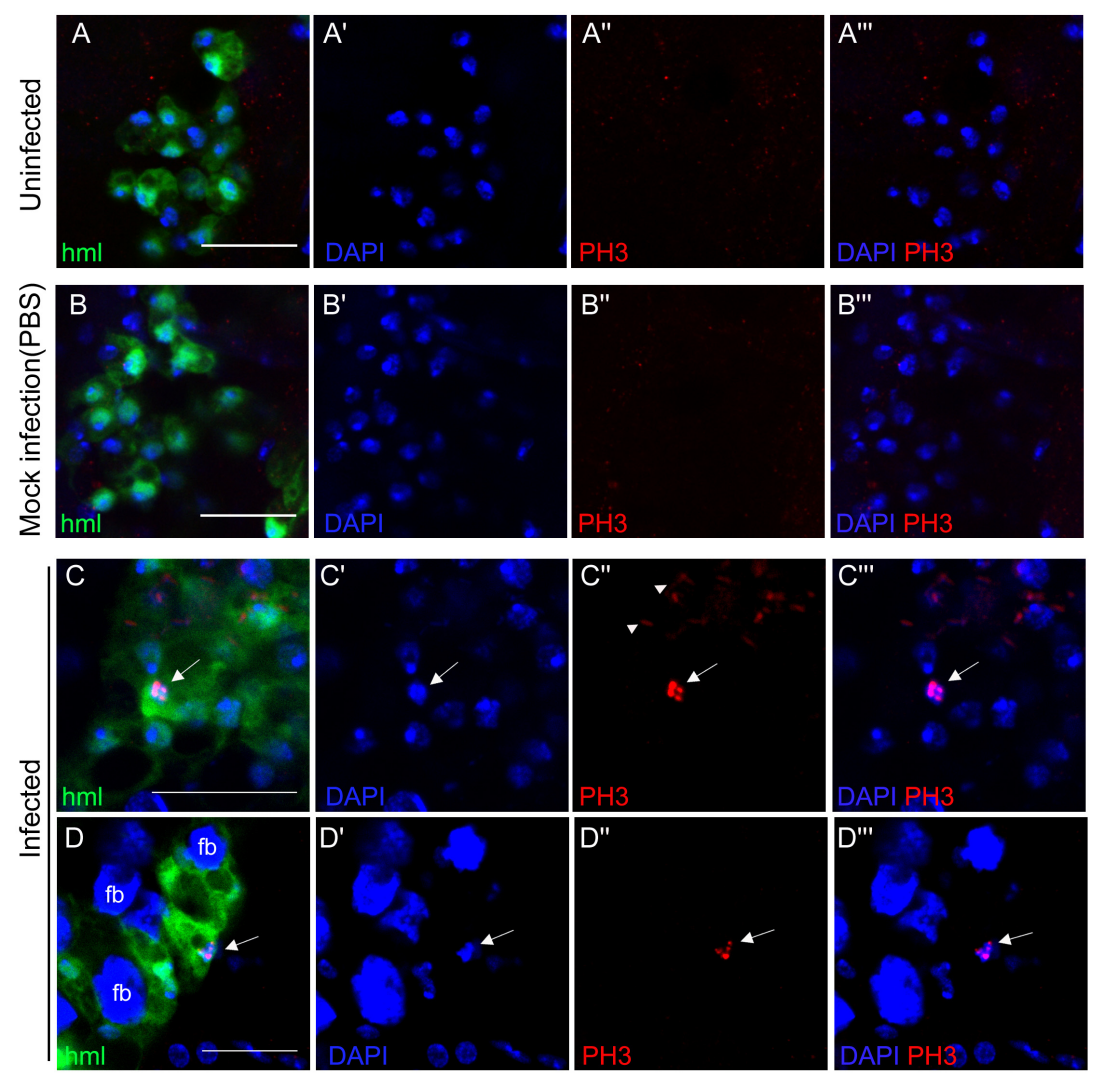
PH3 expression marks the mitotically active adult hemocytes in response to infection. ( **A**– **A’’’**) Control uninfected flies do not show PH3 expression in the Hml positive hub resident hemocytes. ( **B**– **B’’’**) Mock infection with sterile PBS does not evoke a proliferative response from the hemocytes (PH3 ^-^). ( **C**– **C’’’**) and ( **D**– **D’’’**) Two examples from infected adult flies whose hematopoietic hub shows mitotically active (PH3 ^+^) hemocytes (arrow). Arrowhead marks the hemocytes that have engulfed *E.coli.* Scale bar: 20µm.

Employing the above method, no PH3 expression is seen within the hub resident hemocytes of an uninfected individual. Interestingly, mitotically active plasmatocytes (PH3 ^+^) can be seen in the hematopoietic hub of the infected individuals (arrowhead in Figure 6A–D’’)

It is, thus, very clear that the hemocytes present in the hub still retain the capacity of proliferation, whereas employing the same method; we were unable to detect any proliferation in the circulating hemocytes. These observations suggest that the circulating hemocytes in adult fly might have lost the proliferation capacity unlike their larval counterpart ( Anderl *et al*., 2016; Makhijani *et al*., 2011).

## Discussion

Hemocytes play multiple crucial roles in the Drosophila immune response be it embryonic or larval stages. The important mechanisms of cellular immune response provided by hemocytes are phagocytosis, encapsulation, melanization, and coagulation ( Lemaitre & Hoffmann, 2007). Moreover, during systemic infection the hemocytes produce antimicrobial peptide (AMP) and also communicate with other tissues like fat body via the production of cytokines (Upd3, Spz) and TGF-β signaling ( Agaisse *et al*., 2003; Dimarcq *et al*., 1997; Kounatidis & Ligoxygakis, 2012; Shia *et al*., 2009). *Drosophila* larval stage is a preparatory phase that enables the late larvae to achieve certain required volume for metamorphosis ( Ono, 2014). The robust proliferation of all the major cell types including the macrophages/plasmatocytes enables the larvae to reach the required volume. Prior to the identification of hematopoietic hub ( Ghosh *et al*., 2015), it was believed that there is no active site of hematopoiesis in the adult fruit fly ( Evans & Banerjee, 2003; Evans *et al*., 2003; Honti *et al*., 2014; Wang *et al*., 2013). Thus, it was thought that hemocytes from embryonic and larval lineages constitute the adult blood cell repertoire ( Holz *et al*., 2003). It was further inferred that these hemocytes from earlier stages of development lose their proliferative capacity and enter into senescence in adulthood ( Honti *et al*., 2014).

However, if adult fly is unable to produce new blood cells how do they survive basic hazards of life like a bacterial infection that requires a quick and spontaneous immune response? In vertebrates, such threat is tackled by different type of blood cells that get routinely regenerated. In case of bacterial infection the response of the hematopoietic hub is akin to the sessile patches of the larvae. Within first few hours of infection in larvae, the number of hemocytes in the sessile patches are significantly reduced compared to control ( Márkus *et al*., 2009). A similar response is seen in the case of the hub resident hemocytes ( Ghosh *et al*., 2015). Studies on the adult mosquito, on the other hand, reveal that indeed hemocytes can proliferate upon bacterial infection to increase their number ( King & Hillyer, 2012; Sigle & Hillyer, 2016). This raised the possibility that maybe this is true for *Drosophila* adult too. Due to the limitation of the previous protocols, maybe we are missing the phenomenon. Thus, we attempted to come up with an alternate protocol of proliferation assay sensitive enough to document such an event.

The current protocol is efficient and successful in unraveling the proliferation capacity of hemocytes in adult fly which was previously unappreciated. Although the senescence is prevalent in hemocytes of adult, with this efficient method we have been able to identify the rare proliferation events that can be encountered upon bacterial infection. This, in turn, has led to a new understanding that the hemocyte within the hub has not completely lost their proliferative capacity.

Remarkably, with the same BrdU feeding and infection regime, the circulating hemocytes do not demonstrate any proliferative activity. This contrasting observation evokes few interesting possibilities of hemocyte behaviour in response to infection. First possibility is that the hub resident hemocytes post infection can only undergo proliferation. Secondly, after detecting an infection, hemocytes from circulation migrate to the hub and proliferate there. In that case, the hub environment is permissive and mandatory for proliferation. However, there is also a slim chance that soon after infection, a rare fraction of hub resident hemocytes that undergo proliferation in the hub subsequently enters into circulation. The reason why we are unable to detect them with the same protocol might be that the *ex vivo* bleeding technique does not assure 100% analyses of the entire pool of circulating hemocytes. However, the current protocol is sensitive enough to detect the rare events of proliferation happening in the hub.

Thus, the protocol has helped us to differentiate the hub resident hemocytes from the ones in circulation based on their proliferative capacity or the rigidity of senescence.

We envisage that this protocol can also be used to characterize the proliferative responses of hemocytes in various cancerous fly model (leukemia or tumors) ( Dearolf, 1998; Levinson & Cagan, 2016; Petkau *et al*., 2017; Tipping & Perrimon, 2014), viral infections ( Tassetto *et al*., 2017) as well as scenarios of environmental and metabolic stresses during adulthood( Dionne, 2014).

## Data availability

The data underlying this study is available from OSF. Dataset 1: Wellcome Open Research Manuscript 14560: Detecting proliferation of adult hemocytes in *Drosophila* by BrdU incorporation

DOI http://doi.org/10.17605/OSF.IO/8V9XE ( Mandal, 2018)

Data is available under CC0 1.0 Universal

## Supplementary material

Movie S1: Two examples illustrating that the BrdU incorporation is indeed specific to the hemocyte nucleus.

Movie S2: Two more evidences illustrating that the BrdU signal seen within the hemocytes are not a part of any cell that might have been engulfed by the macrophage rather the hemocyte nucleus is positive for it.
