# Supplementary material for: Detecting proliferation of adult hemocytes in Drosophila by BrdU incorporation and PH3 expression in response to bacterial infection
Source: Wellcome Open Res. 2018 Aug 15;3:47. Originally published 2018 Apr 24. [Version 2] doi: 10.12688/wellcomeopenres.14560.2 (PMC5989151; doi:10.12688/wellcomeopenres.14560.2)
Supplement: Peer review history

# Peer review: Referee response for version 2

**DOI:** 10.21956/wellcomeopenres.16056.r33695

**Published:** 2018-10-02

**Authors:** Bruce A. Edgar, Marco Marchetti

**Affiliations:** 1 Huntsman Cancer Institute, University of Utah, Salt Lake City, UT, USA; 2 University of Utah, Salt Lake City, UT, USA

We think the paper has been satisfactorily improved. It’s a useful, unique study and will be a valuable addition to the literature. We think it’s ready for indexing.

We have read this submission. We believe that we have an appropriate level of expertise to confirm that it is of an acceptable scientific standard.

# Peer review: Referee response for version 1

**DOI:** 10.21956/wellcomeopenres.15848.r33211

**Published:** 2018-06-11

**Authors:** Bruce A. Edgar, Marco Marchetti

**Affiliations:** 1 Huntsman Cancer Institute, University of Utah, Salt Lake City, UT, USA; 2 University of Utah, Salt Lake City, UT, USA

Summary:

 The protocol is well written (grammar aside) and quite clear, but it doesn't add much more info than the 2015 paper by the same authors. Indeed, in that publication they had already described all the techniques detailed in this new methods paper, and, albeit they were written in a simpler format, the previously published methods were exhaustive enough to be easily replicated. Hence I don’t see a compelling reason to describe in more detail the methods section of a previous paper, especially considering that the past article is 3 years old.

Minor comments:

- The Grammar needs to be extensively checked;
- Figures 1C and 1D have no purpose since 1C’ and 1D’ are the same but enlarged (and clearer);
- The fly bleeding experiments could use pictures to better describe the experiment;
- Figures 3B-B’-B’’ are not really useful since they do not give more info then 3A and 3C;
- PH3 staining would be good to confirm proliferation;
- Although they are nicely described, the BrdU incorporation, hub stainings, and bleeding procedures were already detailed in their 2015 publication, albeit in a reduced format.

We have read this submission. We believe that we have an appropriate level of expertise to state that we do not consider it to be of an acceptable scientific standard, for reasons outlined above.

# Response

**Published:** 2018-08-02

**Authors:** Lolitika Mandal

**Affiliations:** India Institute of Science Education and Research Mohali, India

Summary: The protocol is well written (grammar aside) and quite clear,

**Thanks for your appreciation and for reviewing our protocol.**

…but it doesn't add much more info than the 2015 paper by the same authors. Indeed, in that publication they had already described all the techniques detailed in this new methods paper, and, albeit they were written in a simpler format, the previously published methods were exhaustive enough to be easily replicated. Hence I don’t see a compelling reason to describe in more detail the methods section of a previous paper, especially considering that the past article is 3 years old.

***Here is where we beg to differ with the Reviewers. The current article is an improved version of one published in Developmental Cell. In fact, because of word limitation, we had to provide a restricted description of the protocol in our previous publication. We felt that a detailed step-by-step narration is essential for easy handling of hematopoietic hub and replication of the assays related to immuno-staining, infection and proliferation in adult fruit fly. Furthermore, the proliferation assay of circulating adult hemocytes presented here is a new data. In the revised version we have also.included the PH3 labeling to endorse the mitotically active state of the hemocytes.***.

Minor comments: The Grammar needs to be extensively checked;

***Taken care off.***

Figures 1C and 1D have no purpose since 1C’ and 1D’ are the same but enlarged (and clearer); ***We have done the necessary changes.***

The fly bleeding experiments could use pictures to better describe the experiment;

***Thank for your suggestion. We have incorporated pictures of adult fly bleeding in Figure 2, in the revised version.***

Figures 3B-B’-B’’ are not really useful since they do not give more info then 3A and 3C;

***We have changed it accordingly in the revised version.***

PH3 staining would be good to confirm proliferation;

***Thank you so much for raising the point. We have performed the experiment to mark the mitotic phase of cell cycle by PH3 staining.  The image and the protocol are incorporated in the revised manuscript (Figure 6 and steps in ‘Colchicine feeding of adult fly and PH3 staining’).***

Although they are nicely described, the BrdU incorporation, hub stainings, and bleeding Procedures were already detailed in their 2015 publication, albeit in a reduced format.

***We are thankful to all the reviewers for their constructive suggestions. The description in the publication is not elaborate. We felt the necessity to provide a step-by-step detail protocol of experiments to provide a proper replication of the results for future research. In addition, the revised manuscript also includes new protocol of hemocyte proliferation assay by PH3 expression using colchicine treatment.***

# Peer review: Referee response for version 1

**DOI:** 10.21956/wellcomeopenres.15848.r33207

**Published:** 2018-06-01

**Authors:** István Ando, Viktor Honti

**Affiliations:** 1 Immunology Unit, Institute of Genetics, Biological Research Centre, Hungarian Academy of Sciences, Szeged, Hungary; 2 Biological Research Centre of the Hungarian Academy of Sciences, Szeged, Hungary

The article provides sufficient information for reproduction of the experiments. The data are well documented.

 Comments:

 The conclusions on the cell division could be corroborated by using an independent marker (e.g. PHH3) for cell division. BrdU is incorporated into the newly synthetized strand of the DNA, however BrdU incorporation does not necessarily reflect division ^1^^-^^7^.

 It was suggested previously by the same authors ^8^that cells from the hub enter the circulation “soon after infection there is a quick release of a large fraction of hemocytes from the hub ( Figures S4C–S4F) highlights the contribution of the hub to fight out infection”. In this case we would expect BrdU labelled cells entering the circulation in the course of the five day labelling. The reason for this seemingly paradox situation should be discussed.

 To draw the conclusion on the effect of bacterial induction, data obtained from a control group, injected with bacterium free buffer, should be presented.

 In general, the technical part of the submission is appropriate, however the conclusions should be strengthened.

We have read this submission. We believe that we have an appropriate level of expertise to confirm that it is of an acceptable scientific standard.

## References

[1] BreunigJJArellanoJIMacklisJDRakicP: Everything that glitters isn't gold: a critical review of postnatal neural precursor analyses. *Cell Stem Cell*.2007;1(6) : 10.1016/j.stem.2007.11.008 612-27 10.1016/j.stem.2007.11.008 18371403

[2] CaldwellMAHeXSvendsenCN: 5-Bromo-2'-deoxyuridine is selectively toxic to neuronal precursors in vitro. *Eur J Neurosci*.2005;22(11) : 10.1111/j.1460-9568.2005.04504.x 2965-70 10.1111/j.1460-9568.2005.04504.x 16324131

[3] DuqueARakicP: Different effects of bromodeoxyuridine and [3H]thymidine incorporation into DNA on cell proliferation, position, and fate. *J Neurosci*.2011;31(42) : 10.1523/JNEUROSCI.3092-11.2011 15205-17 10.1523/JNEUROSCI.3092-11.2011 22016554PMC3225276

[4] GoldsworthyTLDunnCSPoppJA: Dose effects of bromodeoxyuridine (BRUD) on rodent hepatocyte proliferation measurements. *Toxicologist*.1992;12(265) :

[5] KuanCYSchloemerAJLuABurnsKAWengWLWilliamsMTStraussKIVorheesCVFlavellRADavisRJSharpFRRakicP: Hypoxia-ischemia induces DNA synthesis without cell proliferation in dying neurons in adult rodent brain. *J Neurosci*.2004;24(47) : 10.1523/JNEUROSCI.3883-04.2004 10763-72 10.1523/JNEUROSCI.3883-04.2004 15564594PMC2590933

[6] QuTYDongXJSugayaIVaghaniAPulidoJSugayaK: Bromodeoxyuridine increases multipotency of human bone marrow-derived stem cells. *Restor Neurol Neurosci*.2004;22(6) :459-68 15798364

[7] YangYGeldmacherDSHerrupK: DNA replication precedes neuronal cell death in Alzheimer's disease. *J Neurosci*.2001;21(8) :2661-8 1130661910.1523/JNEUROSCI.21-08-02661.2001PMC6762514

[8] GhoshSSinghAMandalSMandalL: Active hematopoietic hubs in Drosophila adults generate hemocytes and contribute to immune response. *Dev Cell*.2015;33(4) : 10.1016/j.devcel.2015.03.014 478-88 10.1016/j.devcel.2015.03.014 25959225PMC4448147

# Response

**Published:** 2018-08-02

**Authors:** Lolitika Mandal

**Affiliations:** India Institute of Science Education and Research Mohali, India

The article provides sufficient information for reproduction of the experiments. The data are well documented.

***Thank you so much for your comments and appreciation.***

Comments: The conclusions on the cell division could be corroborated by using an independent marker (e.g. PHH3) for cell division. BrdU is incorporated into the newly synthetized strand of the DNA, however BrdU incorporation does not necessarily reflect division .

***Thank you so much for raising the point. We understand the genuine concern of reviewer. We have performed the experiment to mark the mitotic phase of cell cycle by PH3 staining. As we have noticed in BrdU incorporation assay (both in this manuscript and in our previous article on adult hematopoietic hub), the number of adult hemocytes undergoing replication is very less and the response is random. Considering the fact that the duration of M phase is shorter when compared to S phase in cell cycle, we adopted a strategy to mark the adult hemocytes that are in the M phase. We reared adult fly in food supplemented with colchicine (colchicine blocks the microtubule polymerisation in cells and thus arrest the dividing cells in different stages of mitosis) to arrest the rarely dividing cells in mitosis. This step increases the probability of tracing the mitotically active hemocytes upon bacterial infection (by PH3 labelling). Using this protocol(1mg/ml concentration of colchicine), we have successfully demonstrated the Hml GFP positive PH3 positive adult hemocytes undergo proliferation in response to bacterial infection. The images and the protocol are incorporated in the revised manuscript (Figure 6 and steps in ‘Colchicine feeding of adult fly and PH3 staining’).***

It was suggested previously by the same authors that cells from the hub enter the circulation “soon after infection there is a quick release of a large fraction of hemocytes from the hub (Figures S4C–S4F) highlights the contribution of the hub to fight out infection”. In this case we would expect BrdU labelled cells entering the circulation in the course of the five day labelling. The reason for this seemingly paradox situation should be discussed.

***Thank you for raising this point. We have discussed the different possible outcome of the result. It might very well be possible that after detecting an infection, hemocytes migrate to the hub and proliferate. In that case, the hub environment permits proliferation. There is also  a slim chance that soon after infection, a rare fraction of hub resident hemocytes that undergo proliferation in the hub subsequently enters into circulation. The reason why we are unable to detect them with the same protocol might be that the ex vivo bleeding technique does not assure 100% analyses of the entire pool of circulating hemocytes. However, we want to draw your attention that the sensitivity of the current protocol allows the rare events of proliferation to be documented in case of the hematopoietic hub.***

To draw the conclusion on the effect of bacterial induction, data obtained from a control group, injected with bacterium free buffer, should be presented.

***We have performed control mock infection experiment earlier and now have incorporated in the revised manuscript. We have mentioned the step of control experiment at point 4. “As a control experiment a mock infection was performed. Similarly aged thorax of an adult flies are pierced with fine glass capillary dipped in a sterile 1X PBS (similar to point 2)  and the flies are subjected to all the steps similar to the experimental flies).***

In general, the technical part of the submission is appropriate, however the conclusions should be strengthened.

***We have worked on to the conclusion part in the revised version.***

***Deeply appreciate your inputs and comments in enriching our work.***

# Peer review: Referee response for version 1

**DOI:** 10.21956/wellcomeopenres.15848.r33030

**Published:** 2018-05-18

**Authors:** Edan Foley

**Affiliations:** 1 Department of Medical Microbiology and Immunology, University of Alberta, Edmonton, AB, Canada

SUMMARY

*Drosophila *is routinely used for the study of blood cell development, migration, and function. Until recently, there was a general assumption that hemocytes were non-proliferative in the adult. However, a recent study ^1^ showed the existence of proliferative hemocytes within the adult hematopoietic hub. This manuscript presents a straightforward procedure to isolate and visualize the hub from adult *Drosophila*. The protocol is well-written and easy to follow. The method should be easy for interested group to replicate, and is a valuable addition to tools for studying blood cells in *Drosophila*.

 Most of my comments are relatively minor.

1. The title only discusses proliferation. However, the data and protocol show how this method can be used to visualize phagocytosis and proliferation. I recommend that the authors amend the title accordingly.
2. The hematopoietic hub is poorly defined in the introduction. The authors should consider a brief summary of what we know about the hub.
3. There is a tendency to verbose statements “resounding similarities” “huge number”, “any infection”, “majorly blocked” “huge infection load” “throwing numerous filopodial extensions” “Majorly all cell types” “a major number of macrophages”. I encourage the authors to consider a more measured approach.
4. In the introduction, it would be helpful to acknowledge that hemocytes also have effector roles (e.g. antimicrobial peptide production) and signaling roles (e.g. release of upd cytokines).
5. It is not clear what is meant by “phases” (abstract), and “tumorigenic conditions” (abstract and discussion).
6. In the section dealing with feeding BrdU to adults, the following sentence is difficult to follow: “The feeding of BrdU will generate a pool of modified nucleotide base in the system”. I recommend the authors re-write this sentence to clarify that BrdU is incorporated in DNA during S phase, and can be detected using commercially available anti-BrdU antibodies.
7. The section dealing with Bacterial infection of the adult fly is missing an important control. Did the authors do a mock infection lacking *E. coli*? If so, they should include a sentence clarifying how to do this. Step 4 should clarify that the anti-BrdU antibody marks replicating cells, including hemocytes.
8. The authors should provide details on the *E. coli *strain used in this study, and on the vendor and catalog numbers required for fine surgical forceps and scissors.
9. I’m concerned by the claim in the results section that circulating hemocytes “must have lost” proliferating capacity. Aren’t alternative explanations possible? For example, isn’t it possible that circulating hemocytes detect infection, migrate to the hub, and start to divide? In this case, a circulating hemocyte still has proliferative capacity. The authors may well be correct with their assumption, but I recommend replacing “must” with slightly less definitive terminology.
10. The imaging section is short. More details are needed about how images are processed in ImageJ and Imaris.
11. Minor comment: “was further testified” should read “was confirmed”
12. What is the evidence that adult flies “lose a major number of macrophages during infection”? In the discussion, it would be helpful to briefly cover what we know about the roles of hemocytes in the immune response of *Drosophila*. There are several studies that looked at this question.

I have read this submission. I believe that I have an appropriate level of expertise to confirm that it is of an acceptable scientific standard.

## References

[1] GhoshSSinghAMandalSMandalL: Active hematopoietic hubs in Drosophila adults generate hemocytes and contribute to immune response. *Dev Cell*.2015;33(4) : 10.1016/j.devcel.2015.03.014 478-88 10.1016/j.devcel.2015.03.014 25959225PMC4448147

# Response

**Published:** 2018-08-02

**Authors:** Lolitika Mandal

**Affiliations:** India Institute of Science Education and Research Mohali, India

SUMMARY :Drosophila is routinely used for the study of blood cell development, migration, and function. Until recently, there was a general assumption that hemocytes were non-proliferative in the adult. However, a recent study showed the existence of proliferative hemocytes within the adult hematopoietic hub. This manuscript presents a straightforward procedure to isolate and visualize the hub from adult Drosophila. The protocol is well-written and easy to follow. The method should be easy for interested group to replicate, and is a valuable addition to tools for studying blood cells in Drosophila.

***Thank you so much for summarising the manuscript and for your appreciation.***

Most of my comments are relatively minor. The title only discusses proliferation. However, the data and protocol show how this method can be used to visualize phagocytosis and proliferation. I recommend that the authors amend the title accordingly.

***Thank you for the suggestion. We have revised the title accordingly. The revised title is: ‘Detecting proliferation of adult hemocytes in Drosophila by BrdU incorporation and PH3 expression in response to bacterial infection’***

The hematopoietic hub is poorly defined in the introduction. The authors should consider a brief summary of what we know about the hub.

***Thank you for your input. We have now described the hematopoietic hub adequately in the introduction of the revised version.***

There is a tendency to verbose statements “resounding similarities” “huge number”, “any infection” “majorly blocked” “huge infection load” “throwing numerous filopodial extensions” “Majorly all cell types” “a major number of macrophages”. I encourage the authors to consider a more measured approach.

***We have changed the phrases accordingly in the respective sentences.***

In the introduction, it would be helpful to acknowledge that hemocytes also have effector roles (e.g. antimicrobial peptide production) and signaling roles (e.g. release of upd cytokines). Thank you for your input.

***We have incorporated more about the effector functions of hemocytes in the introduction as well as the discussion section.***

It is not clear what is meant by “phases” (abstract), and “tumorigenic conditions” (abstract and discussion).

***Sorry for the confusion created.***

***We have mentioned the phases (primitive and definitive hematopoiesis) and tumorigenic conditions (leukaemia and solid tumor) that might induce blood cell proliferation which could be detectable using the current protocol. This is now incoporated both in absract and discussion of the revised version.***

In the section dealing with feeding BrdU to adults, the following sentence is difficult to follow: “The feeding of BrdU will generate a pool of modified nucleotide base in the system”. I recommend the authors re-write this sentence to clarify that BrdU is incorporated in DNA during S phase, and can be detected using commercially available anti-BrdU antibodies.

***Thank you for mentioning the point. Now we have clarified the role of BrdU in detection of cell proliferation. Following description has been included in the method section: “Incorporation of Bromodeoxyuridine (BrdU), a thymidine analog is an established assay for determining cell proliferation in different organisms. Replicating cells during the S phase of cell cycle readily incorporates BrdU instead of thymidine. (Rearing flies in BrdU food generates a pool of BrdU in the system that increases the chance of incorporation in cells specially the ones that undergo rare cell division).The BrdU incorporation can be detected by commercially available anti-BrdU antibody”***

The section dealing with Bacterial infection of the adult fly is missing an important control. Did the authors do a mock infection lacking E. coli? If so, they should include a sentence clarifying how to do this. Step 4 should clarify that the anti-BrdU antibody marks replicating cells, including hemocytes.

***A mock infection was always done with the experimental batches.***

***We are sorry for not mentioning the details regarding it in the previous version. We have incorporated it into the revised version of the manuscript (‘Bacterial infection in adult fly’ at point 4. “As a control experiment a mock infection was performed. Similarly aged thorax of an adult flies are pierced with fine glass capillary dipped in a sterile 1X PBS (similar to point 2)  and the flies are subjected to all the steps similar to the experimental flies).***

The authors should provide details on the E. coli strain used in this study, and on the vendor and catalog numbers required for fine surgical forceps and scissors.

***The E.coli strain used in our study is DH5α. Although the procedures discussed in the manuscript can be efficiently replicated using scissors and forceps obtained from Fine Science Tools (FST). We have used the fine forceps and scissors obtained from a local surgical store.***

I’m concerned by the claim in the results section that circulating hemocytes “must have lost” proliferating capacity. Aren’t alternative explanations possible? For example, isn’t it possible that circulating hemocytes detect infection, migrate to the hub, and start to divide? In this case, a circulating hemocyte still has proliferative capacity. The authors may well be correct with their assumption, but I recommend replacing “must” with slightly less definitive terminology.

***We understand your concern and do agree with your interpretation to a extent. It might very well be possible that after detecting an infection, hemocytes migrate to the hub and proliferate. In that case, the hub environment permits proliferation. There is also  a slim chance that soon after infection, a rare fraction of hub resident hemocytes that undergo proliferation in the hub subsequently enters into circulation. The reason why we are unable to detect them with the same protocol might be that the ex vivo bleeding technique does not assure 100% analyses of the entire pool of circulating hemocytes. However, we want to draw your attention that the sensitivity of the current protocol allows the rare events of proliferation to be documented in case of the hub.***

The imaging section is short. More details are needed about how images are processed in ImageJ and Imaris.

***We have incorporated in details the step-by-step procedure both for ImageJ and Imaris software mentioned in ‘Imaging section’.*** ****

Minor comment: “was further testified” should read “was confirmed”

***We have reframed the sentence with the advised phrase in the result section.***

What is the evidence that adult flies “lose a major number of macrophages during infection”?

***Our analyses of the hematopoietic hub revealed that soon after infection there is a rapid release of macrophages from the hub. In such a scenario, the response of the hematopoietic hub is akin to the sessile patches of the larvae. Within first few hours of infection the number of hemocytes in the sessile patches are significantly reduced compared to control (Markus et al., 2009). Based on the fact that there is loss of the resident hemocytes in both cases, we made that inference.We have explained this in the revised version.***

*In the discussion, it would be helpful to briefly cover what we know about the roles of hemocytes in the immune response of Drosophila. There are several studies that looked at this question.*

***We have incorporated different roles of hemocyte in immune response in the revised manuscript. Thanks for your inputs.***
